# The Effect of Ageing on Ocular Blood Flow, Oxygen Tension and Retinal Function during and after Intraocular Pressure Elevation

**DOI:** 10.1371/journal.pone.0098393

**Published:** 2014-05-27

**Authors:** Jeremiah K. H. Lim, Christine T. O. Nguyen, Zheng He, Algis J. Vingrys, Bang V. Bui

**Affiliations:** Department of Optometry and Vision Sciences, The University of Melbourne, Victoria, Australia; Hanson Institute, Australia

## Abstract

**Purpose:**

To investigate the effect of ageing on the recovery of ocular blood flow, intravitreal oxygen tension and retinal function during and after intraocular pressure (IOP) elevation.

**Methods:**

Long Evans rats (3- and 14-month-old) underwent acute stepwise IOP elevation from 10 to 120 mmHg (5 mmHg steps each 3 minutes). IOP was then returned to baseline and recovery was monitored for 2 hours. Photopic electroretinograms (ERG) were recorded at each IOP step during stress and at each minute during recovery. Ocular blood flow and vitreal oxygen tension (pO_2_) were assayed continuously and simultaneously using a combined laser Doppler flow meter (LDF) and an oxygen sensitive fibre-optic probe, respectively. The combined sensor was placed in the vitreous chamber, proximal to the retina. Data were binned into 3 minute intervals during stress and 1 min intervals during recovery. Recovery data was described using a bi-logistic function.

**Results:**

Rats of both ages showed similar susceptibility to IOP elevation, with pO_2_ showing a closer relationship to ERG than LDF. During recovery, both ages showed a distinctive two-phased recovery for all three measures with the exception of the LDF in 3-month-old rats, which showed only 1 phase. In all animals, LDF recovered fastest (<1 minute), followed by pO_2_ (<10 minute) and ERG (>1 hour). 14-month-old rats showed surprisingly faster and greater LDF recovery compared to the younger group, with similar levels of pO_2_ recovery. However, the ERG in these middle-aged animals did not fully recover after two hours, despite showing no difference in susceptibility to IOP during stress compared to the young group.

**Conclusions:**

Young and middle-aged eyes showed similar susceptibility to IOP elevation in terms of pO_2_, LDF and ERG. Despite this lack of difference during stress, older eyes did not completely recover function, suggesting a more subtle age-related susceptibility to IOP.

## Introduction

Ageing is considered a major risk factor in many neurodegenerative diseases, including Parkinson's and Alzheimer's disease [1]. In the eye, the neurodegenerative disease glaucoma is strongly associated with senescence, an observation that cannot be accounted for by only small age-related increases in intraocular pressure (IOP). This raises the possibility that some inherent neuronal, structural or vascular susceptibility [2], places older eyes at greater risk of IOP-related injury.

Changes to energetic factors such as mitochondrial dysfunction [3], blood flow regulation and oxygen usage have been suggested to underlie age-related susceptibility to injury. In particular, retinal blood flow has been shown to decline with age, a phenomenon that has been attributed to increased vascular resistance [4]–[8], decreased metabolic consumption due to age-related cell loss [9], rheological changes [10] in blood vessels and increasing IOP with age [11], [12]. These age-related processes have been reported to begin as early as middle-age in human [13] and laboratory species [14]. Increased vascular resistance and an imbalance in endothelium-derived vasodilatation factors with ageing may also contribute to altered autoregulation and blood flow during stress induced by IOP elevation [15] and recovery [16], [17]. Impairment in the ability for blood flow to regulate during and after IOP elevation may lead to slower functional restoration [18], [19].

Blood flow has been shown to be sensitive to IOP increases beyond 30 mmHg in humans [20]–[22] and rats [23]. In rats, retinal function as measured using the photopic b-wave showed little change until IOP was raised beyond 60 mmHg [24]. On this note, it has been shown that oxygen tension measurements in the vitreous [25], retina [26] and optic nerve [27] showed no appreciable change until IOP was increased to levels above 50–60 mmHg in various species. This suggests the possibility that oxygen tension shares a closer relationship to retinal function than ocular blood flow changes during stress. This close relationship has been corroborated by studies employing simultaneous measurements of oxygen tension and function [28], [29] during ischemia. As such, one might expect this to be the case during recovery from stress.

Indeed, it has been shown that the recovery of ocular blood flow after acute IOP elevation is faster than functional response recorded using the electroretinogram (ERG). These studies show that following 30 minutes of IOP elevation at 80 mmHg, complete blood flow recovery occurs within the first few minutes [30], whereas the ERG does not fully recover until after 60 minutes [17]. One way to fully quantify this relationship is to simultaneously assay ocular blood flow, oxygen tension and the ERG.

Additionally, it is still not known if ageing plays a role in modifying the relationship between these measures. Previous literature has shown that functional changes occur in rats as early as 18 months of age [31]. By choosing an age where functional decline is not seen in rodent eyes, we hope to determine if metabolic changes precede that of functional changes. This is achieved by assaying the rate at which ocular blood flow, vitreal oxygen tension and function varies during IOP elevation and following recovery in a group of young (3-month-old) and middle-aged (14-month-old) animals.

## Materials and Methods

### Animals

All experimental procedures abide by the National Health and Medical Research Council Australian Code of Practice for the care and use of animals for scientific purposes. Ethic approval (0705158.1) was obtained from the Animal Ethic Committee of the Science Faculty in the University of Melbourne. Male Long-Evans rats were housed in a 12 hour light (50 lux maximum) - 12 hour dark cycle. Food and water were made available *ad libitum*. Two cohorts of male Long-Evans rats were assessed, 3-month-old (n = 14) and 14-month-old (n = 16). All experimental procedures were conducted under general anaesthesia of ketamine and xylazine (60∶5 mg/kg intramuscular injection; Troy laboratories Pty Ltd., Smithfield, NSW, Australia). Topical application of proxymetacaine hydrochloride (Ophthetic 5 mg/ml, Allergan, Frenchs Forest, NSW, Aust) and 0.5% tropicamide (Mydriacyl 5 mg/ml, Alcon laboratories, Frenchs Forest, NSW, Aust) provided corneal anaesthesia and mydriasis, respectively.

### Electroretinography

Light-adapted electroretinograms were recorded using custom-made chlorided silver electrodes. The active was placed directly onto the apex of the cornea. The ring-shaped reference was placed behind the limbus around the sclera, while the ground electrode (F-E2-30; Grass Telefactor, West Warwick, RI) was inserted subcutaneously into the tail. Carmellose sodium (Celluvisc 10 mg/mL; Allergan, Irvine CA) was used to maintain corneal hydration throughout the procedure and to facilitate electrode contact with the corneal surface.

The light stimulus of 3 ms duration was delivered using light emitting diodes (8 Watt Luxeon LED, Philips Lumileds Lighting Company, CA, USA) embedded in a Ganzfeld integrating sphere (Photometric Solutions International, Huntingdale, VIC, Australia). To achieve light adaptation, rats were exposed to a constant background luminance of 15 cd/m^2^ for 10 minutes [32] and a stimulus of 2.03 cd·s·m^−2^ was used to elicit a photopic ERG waveform.

Signals were band pass filtered (0.3 to 1000 Hz) and digitized at 4 kHz with 1000x amplification. An additional low pass filter was applied (Blackman filter, 37 Hz, -3 dB) to remove contamination of the rising limb of the b-wave by oscillatory potentials. The photopic ERG b-wave amplitude was then taken from the trough of the a-wave to the peak of the b-wave. During IOP elevation, the ERG was sampled at 20 repeats at 2 second intervals at each IOP step and averaged. During recovery, the ERG waveforms were also collected at 2 second intervals but were analysed individually (no averaging) for the first two minutes. This was done to capture the initial rapid recovery of the ERG. From the second minute onwards, the ERG was again analysed by averaging 20 recordings for each minute. Whilst averaging helped to reduce high frequency noise, analysis of individual waveforms throughout the recovery period did not change the recovery characteristics reported below.

### Ocular blood flow and vitreal oxygen tension

Ocular blood flow and vitreal (free) oxygen tension were measured using a combined fibre-optic sensor (BF/OF/E, Oxford Optronix Ltd, London, UK) placed within the vitreous chamber and close to the retinal surface. In order to insert the probe into the vitreous chamber, a small incision was made 2 mm posterior to the limbus using a 19G needle secured with micromanipulator. The tip of the needle was then inserted to a depth of 1 mm (∼ less than half the bevel length) to create an opening just wide enough to insert the probe (∼23G) along the inside of the 19G cannula. Once the probe was in place the 19G cannula was retracted. The 23G probe does not appreciably displace the lens of the rat eye. The position of the probe was confirmed visually with a microscope (MZ6, Leica microsystems, North Ryde, NSW, Australia). Under the microscope the probe was guided away from the major blood vessels. This was also aided by avoiding locations where pO_2_ was greater than 50 mmHg, indicating a location directly above a major vessel. The probe was advanced until contact with the retinal surface was evidenced by an abrupt decrease in oxygen tension (probe gives off an erroneous negative pO2 value [−200 mmHg] when this occurs due to pressure on the tip). The probe was then retracted by ∼0.25 mm using the micromanipulator, restoring pO_2_ readings to physiological levels within one second.

Another indicator of probe distance to the retina is backscatter, which when <1500 indicates that the probe is just above the retina (see Figure S1). Throughout our experiments average backscatter was relatively stable for both young (one-way ANOVA, F_19,218_ = 0.55, P = 0.94) and older animals (one-way ANOVA, F_19,252_ = 0.20, P>0.99) suggesting that that probe position was stable (see Figure S4).

One limitation of our application of this method, is that we cannot determine the exact distance of the probe from the retina. While retinal imaging and OCT would have helped determine exact probe distance, our probe insertions and preparations for ERG, made imaging very challenging. However the same procedure was conducted in young and old eyes and thus the positioning of the probe is unlikely to influence any age-related effects.

Fibre optic sensors, also known as “optodes” assay the partial pressure of oxygen by measuring the amount of free oxygen directly at the probe tip (230 to 250 µm diameter). This is achieved by measuring fluorescence subsequent to oxygen quenching over a given period of time (fluorescent lifetime) after the fluorophore is excited by a short pulse of light [33], [34]. The probe employed in this study contains a platinum octaethylporphyrin fluorophore immobilised within a silicone rubber matrix, fixed into the sensor tip. It is excited by a light-emitting diode producing short pulses of blue-green light at 525 nm, which is transmitted through the optical fibre to the probe tip.

Fluorescence decays subsequent to collisions between oxygen and platinum molecules. This oxygen “quenching” of fluorescence is described by the Stern-Volmer equation [35], [36] where the lifetime of the fluorescence is inversely proportional to the pO2 in the tissue directly below the probe tip. The lifetime of the fluorescence is then monitored by the OxyLab pO2 receiver (Oxford Optronix, England, UK) which converts readings to pO2 in mmHg according to the Stern-Volmer equation. We did not preheat the room temperature Hanks solution prior to its introduction. However, the particular oxygen sensor (BF/OF/E Oxford Optronix Ltd, London, UK) used has a built in thermocouple which makes real time temperature corrections for variations from 30°C to as high as 44°. With the introduction of small volume, any temperature variation should be small and similar across the two age groups.

A probe placed proximal to the retinal surface is believed to accurately reflect inner retinal oxygen tension [37]–[41]. This was confirmed in rats, where oxygen tension measurements in the vitreous using oxygen sensitive microelectrodes were similar to measurements performed at the inner limiting membrane [41], [42]. While these studies used fine tipped electrodes (1–5 µm), the fibre-optic probe employed in this study has a larger diameter (230–250 µm), and thus averages oxygen tension across a larger surface area of retina. This is likely to provide a better correlate to our global measures of retinal function. Whilst we did not measure peripheral oxygen saturation at the time, we have confirmed that peripheral oxygen saturation (Vetsens, Hornsby, NSW, Aust) remained stable throughout an equivalent period of anaesthesia in adult Long-Evans rats (please see Figure S3) with normal blood pressure (103±6 mmHg) Peripheral oxygen saturation (spO_2_) at baseline was 93% and showed little change over a period of three hours (one-way ANOVA, F_12,36_ = 0.55, P = 0.87). Also as we express our data relative to the animal's starting baseline, small differences in peripheral oxygen saturation may have less of an impact on our data.

The laser Doppler flowmetry (LDF) measurement via the vitreous chamber represents a weighted average of retinal and choroidal blood flow as the probe samples 1 mm^3^ of tissue [43], [44]. While there is no current agreement in the literature regarding the exact ratio of inner to outer retinal blood supply, there is evidence to show that the LDF reflects inner retinal blood flow (He et al, 2012 - online supplement Figure S3 [23]). Others suggest that the retinal pigment epithelium limits the penetration of the laser into the choroid, thus limiting choroidal contribution to the superficial layers [45]. Given the contribution from both retinal and choroidal layers, the terminology ‘ocular blood flow’ is used here. Whilst the application of the LDF in this study has its limitations, we are not aware of a viable alternative measure that be used simultaneously with oxygen tension measurement and the electroretinogram.

Ocular blood flow and vitreal oxygen tension were recorded continuously at a sampling rate of 1 kHz during the 1 hour of step-wise IOP elevation, and for 2 hours after IOP had been returned to baseline. During IOP challenge, oxygen tension and blood flow were binned into 3 minute epochs to match the duration of each IOP step. In order to match the sampling of the ERG during recovery, oxygen and blood flow data were binned into 2 second epochs in the first 2 minutes and into 1 minute bins after the first 2 minutes. There was an initial fast phase (up to 2–10 minutes) followed by a slower prolonged phase (from 2–10 up to 120 minutes) of recovery for pO_2_ and ERG. As such, data are plotted on a log time axis to aid visualisation of both phases.

### Modelling the dynamics of recovery

**Figure 1:**
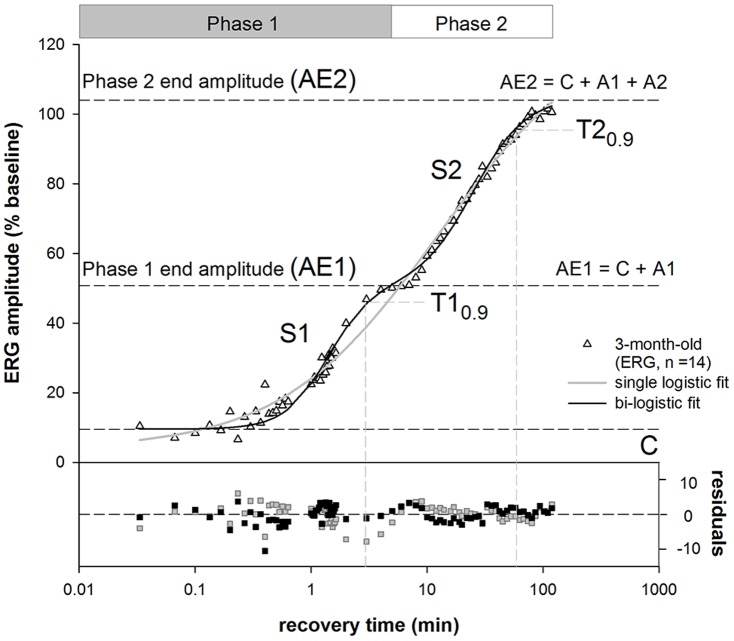
Single and bi-logistic models for recovery of ERG from IOP elevation. Group averaged (n = 14, 3-month-old rats) ERG b-wave amplitudes were plotted against recovery time after IOP was restored to baseline. The bi-logistic function (Equation 2) provides a significantly better fit (black line, F-test, P<0.001, F = 15.80) compared with single logistic function (solid grey line, Equation 1). A is the amplitude of recovery, S is slope of the curve and C is the y-intercept. The suffixes 1 and 2 for each parameter represent the corresponding recovery phases. The time to 90% (T_0.9_, vertical dashed lines) was generated by the model and represents the speed of the recovery at each phase. The end of each phase was returned as the time when the data passed through AE1 or AE2; here this is 6 and 120 minutes. Additional parameters derived from the model include AE1 (A1 + C) which represent the amplitude of phase 1 (black dashed line). AE2 represents the final amplitude (black dashed line, AE2 = A1+A2+C).



The recovery of the ERG, LDF and pO_2_ was modelled with a bi-logistic function [46], to describe the two phase of recovery following acute IOP challenge. This concept is illustrated in Figure 1 for ERG recovery. The need for the bi-logistic function [46] was justified statistically and the residuals show that it provides a superior fit to the data compared with a single logistic [47]. The equation for the single logistic function is given by the following:

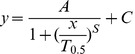
(Equation 1)


Relative recovery (y, %) as a function of time (x, min) is given by its maximum amplitude (A). T_0.5_ is the time taken to achieve half of the maximum amplitude. S is the slope (Δy/Δt) of the curve and C the y-intercept. The bi- logistic function is given by Equation 2:



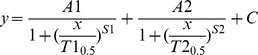
(Equation 2)


Relative recovery (y, %) as a function of time (x, min) is governed by 2 logistic functions. Where A1 is the maximum recovery amplitude of the first phase and A2 the maximum amplitude of the second phase. T1_0.5_ and T2_0.5_ denote the times taken to recover to half of the maximum amplitude in the first and second phases respectively. Meyer [46] has shown that the time to 90% recovery (T_0.9_) is a more robust indicator for full recovery of each phase of the logistic function rather than T_0.5_. As such, the T1_0.9_ and T2_0.9_ were used instead of T_0.5_ in this study (Figure 1). S1 and S2 denote the respective slopes of the recovery phases. Lastly, C denotes the y-intercept.

An F-test was used to compare the goodness of fit of the single and bi-logistic functions. For the three outcome measures (ERG, pO_2_, LDF) and the two age groups (3 and 14 months), the bi-logistic function produced a significantly better fit in 5 out of 6 recovery measures (F-test, P<0.001 for ERG_3,14m_, pO_2 3,14m_ and LDF_14m_). For this reason the bi-logistic function is adopted to model the data. To compare the functions between outcome measure and between ages, a parametised bootstrap [48], [49] was used to determine 95% CI for the parameters of the model. This approach makes no assumptions about the underlying distribution of parameters and return robust confidence limits.

### Intraocular pressure and ocular perfusion pressure

Two approaches can be employed to study the effect of a range of IOP levels on the eye. The first is to employ separate cohorts of animals at one IOP level, which is resource intensive [24]. As LDF is a relative measure this approach does not lend itself to looking at changes due to IOP. An alternative approach to return information across all IOP level is to employ a step-wise protocol, this allows ERG, LDF and pO_2_ to be measure simultaneously during IOP elevation to a range of levels. In addition, recovery can also be assessed immediately after IOP elevation. Intraocular pressure was manipulated via cannulation of the anterior chamber with a 27G needle attached to a pressure transducer (Transpac, Abbott Critical Care Systems, Sligo, Ireland), whose output was captured (Powerlab 8/SP, ADInstruments, Castle Hill, Aust) and displayed with Chart software (ADInstruments). The other side of the pressure transducer was connected to a reservoir containing room temperature and air equilibrated Hanks balanced salt solution (JRH Biosciences, Kansas, KS, USA) at a precalibrated height, which determined the IOP level. This “single needle” with a 27G needle, has relatively low resistance and has been shown to provide robust IOP elevation and accurate IOP determination (He et al, 2012 - online supplement [23]). Initial IOP levels were set at 10 mmHg and IOP was gradually raised to 100 mmHg in steps of 5 mmHg, with each step lasting 3 minutes, followed by a step up to 120 mmHg for another 3 minutes.

The entire stress protocol lasted for 60 minutes. Part of the data was used in another manuscript to develop a mathematical model for ocular perfusion pressure (OPP) challenge [50].

As previously described [23], OPP was calculated using Equation 3.



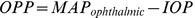
(Equation 3)


This formulation is based on the assumption that IOP approximates venous pressure in the eye, which allows OPP estimation by subtracting the IOP from mean arterial pressure (MAP_ophthalmic_) [51], [52]. Tail cuff sphygmomanometry has been shown to return accurate measures of MAP in anaesthetised rats [53], [54] (also see Figure S2). Whilst this approach is less accurate that blood pressure measure via an indwelling cannula, BP was neither a primary outcome measure nor expected to fluctuate under stable anaesthesia, thus it should be suitable for the purposes of this study [55]. The average systolic blood pressure of the 3-month-old cohort was 98 mmHg±3.8 (SEM) and 97 mmHg±3.3 for the 14-month-old group during IOP elevation. During recovery, average BP was 98 mmHg±6.1 and 94 mmHg±4.3 respectively. Due to differences in baseline BP and IOP, this resulted in animals having different starting OPP values, causing sample size drop offs at the extremes. As such, only the OPPs ranging from 0 to 95 mmHg were analysed.

### Statistics

All group data were expressed as average ± standard error of the mean (SEM) with the exception of bootstrapped data where a non-parametric 95% confidence interval (CI) is shown. To compare response amplitudes as a function of OPP, two-way ANOVA with Bonferroni post-hoc tests were used. Significant difference (P<0.05) in parameters of the bi-logistic function was assumed if the mean of one group fell outside the confidence limits of the other group.

## Results

### ERG during IOP elevation

**Figure 2:**
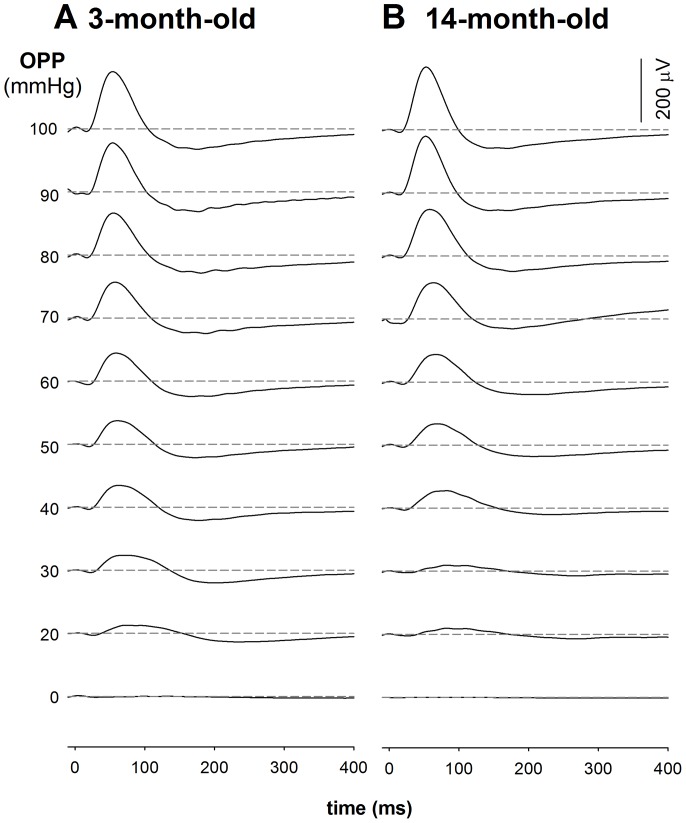
Representative photopic ERG waveforms alter with IOP induced OPP changes in 3-month-old (A) and 14-month-old (B) rats. ERGs were collected after 15 min of light adaptation on a 15/m^2^ background in young (n = 14) and old (n = 16) Long Evans. Panel A shows a typical gradient OPP profile for the ERG in a 3-month-old rat with a baseline blood pressure of 116 mmHg. Panel B shows a typical gradient OPP profile for the ERG in a 14-month-old rat with a baseline blood pressure of 114 mmHg.

Figure 2 shows the effect of stepwise IOP elevation on the photopic ERG in a representative 3- and 14-month old rat. The photopic b-wave decreased as IOP was raised above 60 mmHg. This functional susceptibility appears to be similar between young and older rats during IOP elevation.

### Effect of IOP elevation on blood flow, oxygen tension and function

Prior to IOP elevation, the average (mean ± SEM) absolute values at baseline in 3-month-old rats were LDF 851 A.U. ±347, pO_2_ = 31 mmHg±5 and ERG = 166 µV±13. In the 14-month-old rats, absolute values were LDF 1223 A.U. ±716, pO_2_ = 33 mmHg±3 and ERG = 236 µV±5.

**Figure 3:**
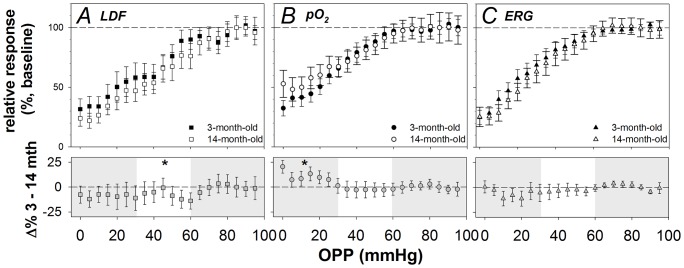
Effects of age on LDF, pO_2_ and ERG during OPP challenge. Simultaneous measurement of blood flow (A), vitreal oxygen tension (B) and function (C) during step-wise IOP elevation in 3-month (n = 14, unfilled symbols) and 14-month-old (n = 16, filled symbols) rats reveal differences in sensitivity between measures (average ± SEM) during stress. Blood flow was most sensitive to OPP challenge, followed by oxygen and ERG, with the latter two parameters showing similar sensitivity. The lower panels show the residuals between the 3- and 14-month-old (3–14 m.o.) for the corresponding measure in the upper panel. The shaded areas indicate the high OPP (OPP: 95–65 mmHg) and low OPP phases (25–0 mmHg) while the unshaded area in between represents the moderate OPP phase (60–30 mmHg). The asterisks denote significance of P<0.05 for the summed residual for the given phase. Older animals showed unexpectedly better blood flow regulation in the moderate OPP range, while younger animals show better oxygen tension regulation in the low OPP range. There was no difference in function during OPP challenge between these age groups.

Figure 3 shows LDF, pO_2_ and ERG relative to baseline plotted as a function of OPP for 3- and 14-month-old rats. When taking into account the full range of OPP, there were no differences in LDF response to OPP lowering between the ages (two-way ANOVA, main effects, F_1,19_ = 2.82, p = 0.09). This was also the case for pO_2_ (F_1,19_ = 1.01, p = 0.32) and ERG (F_1,19_ = 3.39, p = 0.07). None of the parameters showed significant interaction.

When considering the residuals (difference between 3- and 14-month-old) as shown in the lower panels of Figure 3, LDF in the older eyes appeared to be more resistant to OPP reduction than the young group, at moderate OPPs (Figure 3A, lower, P<0.05). When comparing pO_2_, 3-month-old rats showed greater relative oxygen tension levels at lower OPPs (Figure 3B, lower, P<0.05).

### ERG recovery from IOP elevation

**Figure 4:**
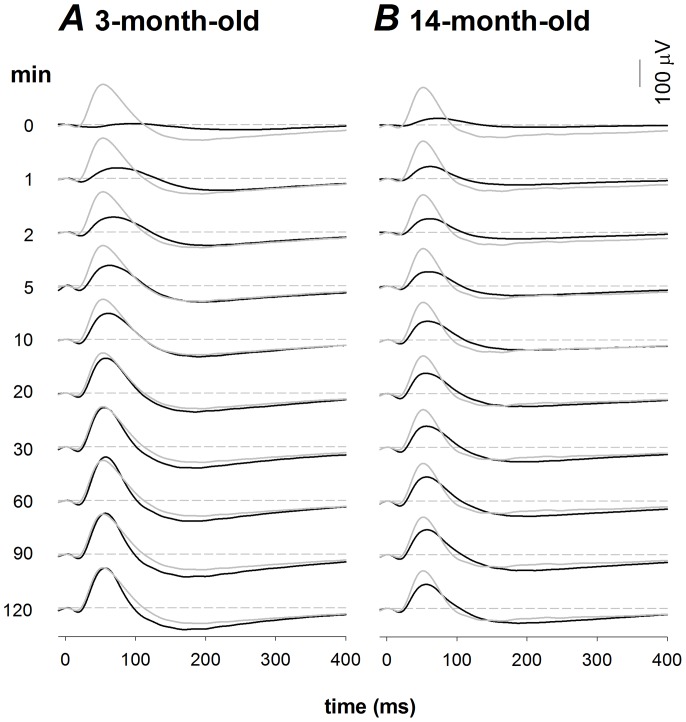
Representative photopic ERG waveforms of a 3-month-old (A) and 14-month-old animal (B) during recovery from acute IOP elevation. The black traces represent the average waveform at each time point whereas the grey waveforms indicate the pre-IOP baseline. The dashed lines indicate zero amplitude for reference.

Figure 4 shows the recovery of the photopic ERG following 1 hour of stepwise IOP elevation in a representative 3- and 14-month-old animal. ERG recovery was rapid during the first 10 minutes, and became more gradual over the next 50 minutes. After the first hour, there appeared to be little further change to the ERG. In the 14-month-old rats, it was common for the ERG to remain below pre-IOP elevation baseline after two hours of recovery. In contrast, most 3-month-old animals recovered back to baseline after two hours.

### Modelling Recovery

**Figure 5:**
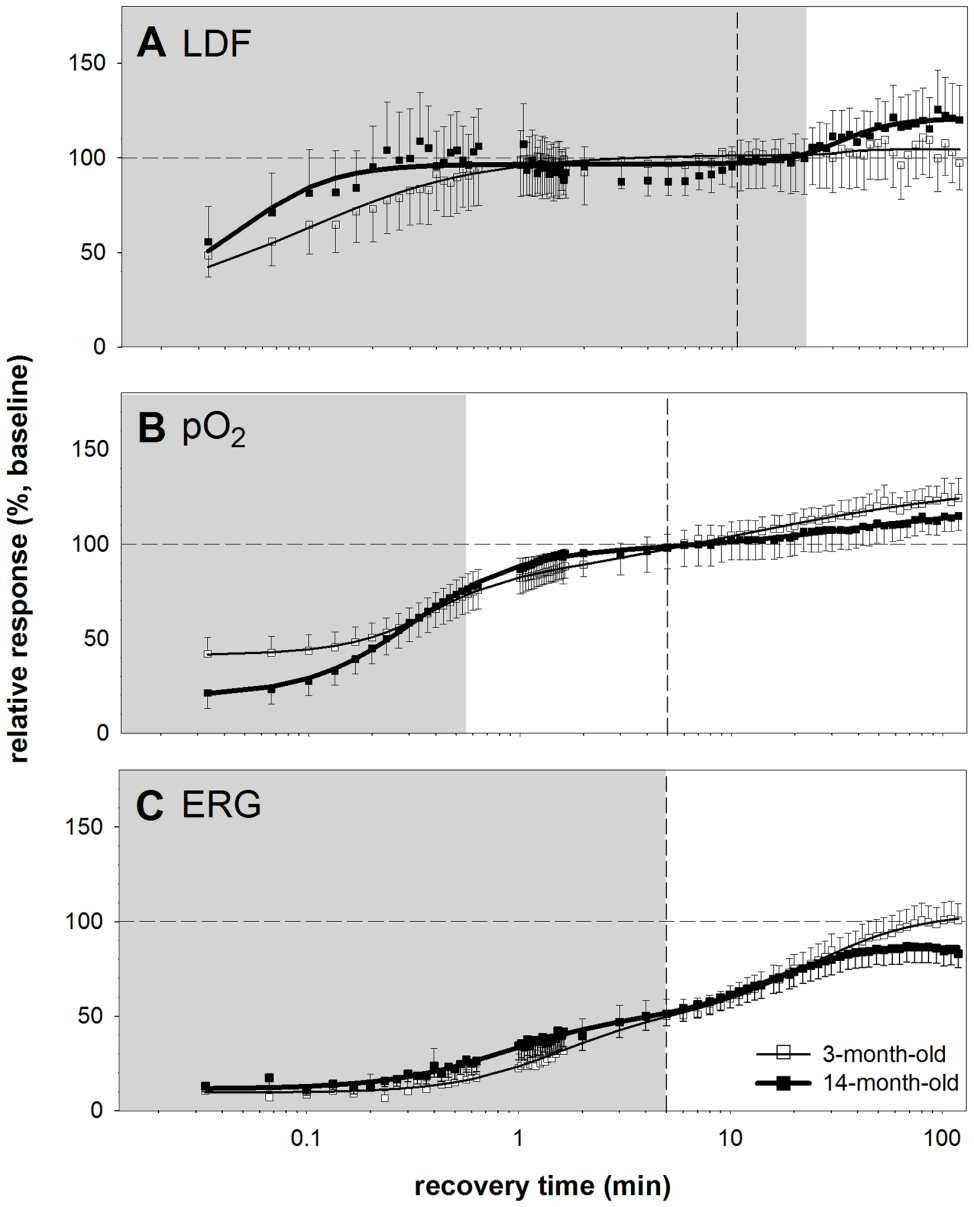
Recovery from IOP elevation. Relative recovery of LDF, pO_2_ and ERG as a function of time following IOP elevation in 3 (n = 14) versus 14 month (n = 16) old rats (mean ± SEM). Panel A shows the recovery of the LDF, while B. shows pO_2_ and C. shows the ERG recovery. A bi-logistic function was used to model the data. The shaded region denotes the duration of phase 1 recovery for the 3-month-old rats as given by the model and the vertical dashed line indicates the duration of phase 1 recovery for the 14-month-old group. Thin and thick solid lines are used to represent the bi-logistic fit to the 3- and 14-month-old animals respectively.

Figure 5 compares the recovery of blood flow, oxygen tension and function between 3- and 14-month old animals when modelled using the bi-logistic function (Equation 2). This returns two recovery phases for each measure. Phase 1 in 3-month-old rats is denoted by the shaded region whereas the vertical dashed lines show its extent in 14-month-old rats. The age-related changes in parameters are described below in brackets (3-month versus 14-month-old, P value) and all confidence limits are summarized in Table 1.

**Table 1:** Summary of recovery parameters between 3- and 14-month-old animals.

	A1	T1_0.9_	S1	C	AE1	A2	T2_0.9_	S2	AE2
	(%)	(min)	(AU)	(%)	(%)	(%)	(min)	(AU)	(%)
LDF_3m_	79	1	−1.1	22	101	3	1.1	−8.3	104
Lower	65	0.5	−1.3	18	85	2	0.6	−10.1	88
Upper	95	1.4	−0.8	27	115	3	2.0	−6.6	118
LDF_14m_	67	↓ 0.13	↑ −2.1	↑ 29	96	24.	33	−3.3	↑ 120
Lower	54	0.10	−2.5	23	81	19	28	−4.0	104
Upper	80	0.20	−1.7	36	112	29	45	−2.6	137
PO2_3m_	34	0.5	−2.4	40	74	60	110	−0.7	134
Lower	27	0.37	−3.0	32	63	48	63	−0.8	116
Upper	41	0.63	−1.9	48	85	72	119	−0.5	150
PO2_14m_	↑ 78	↑ 1.03	↓ −1.7	↓ 19	↑ 97	↓ 23	↓ 42	↑ −1.1	120
Lower	63	0.63	−2.2	15	82	17	26	−1.4	103
Upper	92	1.54	−1.4	24	113	27	68	−0.8	137
ERG_3m_	41	3	−2.3	10	51	54	58	−1.9	105
Lower	33	2	−2.8	8	42	43	39	−2.3	89
Upper	49	4	−1.8	12	59	65	80	−1.5	120
ERG_14m_	39	3	↓ −1.5	11	50	↓ 36	↓ **26**	↑ −2.1	↓ 86
Lower	32	2	−1.9	9	43	29	20	−2.6	75
Upper	47	4	−1.2	14	59	43	36	−1.7	98

Significantly different parameters are bolded, with an arrow indicating either an increase or decrease in the 14-month-old rat, as compared to the 3-month-old rats. The first column gives the parameter and its lower and upper 95% confidence limits.

### Blood flow recovery in 3- and 14-month old animals

Figure 5A shows that in both 3- and 14-month-old rats, blood flow recovered by similar amounts during the first 23 minutes (A1: 79% versus 67%, P>0.05). By the end of the first phase, blood flow had recovered back to baseline in both age groups (AE1: 101% versus 96%, P>0.05). However, in the second phase, 3-month-old rats showed less change compared with the 14-month-old group (A2: 3% versus 24%, P<0.05). As a result, the 14-month-old group animals showed significantly hyperperfusion at the end of two hours (AE2: 104% versus 120%, P<0.05).

Figure 5A also shows that the speed of blood flow recovery (to 90%, T1_0.9_) during phase 1 was significantly slower in the 3-month-old compared with the 14-month-old age group (T1: 1.0 min versus 0.13 min, P<0.05). These data are surprising in that they show faster LDF recovery in middle-age.

### Oxygen tension recovery in 3- and 14-month-old animals

Figure 5B shows that the starting value for oxygen tension was significantly higher in young compared with the older rats (C: 40% versus 19%, P<0.05). This suggests that IOP elevation reduced vitreal oxygen tension in older eyes more than younger eyes. However, at the end of the first phase of recovery (AE1), the 3-month-old group showed significantly less recovery than did the 14-month-old group (AE1: 74% versus 97%, P<0.05). This can be explained by a greater magnitude of pO_2_ recovery in phase 1 of the older group (A1: 34% versus 78%, P<0.05). Given the greater magnitude of recovery in older eyes over the first phase, it was not surprising that the time taken for 90% recovery in the first phase was also prolonged in 14-month-old compared with the 3-month-old animals (T1: 0.50 min versus 1.0 min, P<0.05).

In the second phase of oxygen recovery, the 3-month-old rats showed more recovery than did the 14-month-old rats (A2: 60% versus 23%, P<0.001). As the young animals recovered greater oxygen levels, they took longer than middle-aged rats (T2: 110 min versus 42 min, P<0.05) to achieve 90% recovery. As a whole, by the end of 2 hours of recovery, both groups showed no statistical differences in recovery magnitude (AE2: 134% versus 120%, P>0.05) with both achieving a significant hyperoxic level some 20% to 34% above baseline.

### ERG recovery in 3- and 14-month-old animals

Figure 5C shows that young and middle-aged rats had similar ERG amplitudes (∼ 10% of baseline values, C: 10% versus 11%, P>0.05) at the start of recovery. They showed a similar magnitude of functional recovery in the first phase (A1: 41% versus 39%, P>0.05) and as a result, had similar magnitudes at the end of phase 1 (AE1: 51% versus 50%, P>0.05). The first phase of recovery showed a similar time constant in young and older rats (T1: 3 min versus 3 min, P>0.05).

In the second phase of ERG recovery, the 3-month-old rats recovered more than did the 14-month-old rats (A2: 54% versus 36%, P<0.05). As a result, the older group took significantly less time to achieve 90% recovery in the second phase compared to the young group (T2: 58 min versus 26 min, P<0.05). This was largely due to the fact that by the end of 2 hours of recovery, the older group had not returned to baseline ERG levels (AE2: 105% versus 86%, P<0.05). In comparison, the young cohort fully recovered to its baseline level.

## Discussion

This study is the first to measure posterior segment blood flow, oxygen tension and function (LDF, pO_2_ and ERG) simultaneously whilst manipulating IOP in ageing rat eyes. We show that during OPP challenge, blood flow is most sensitive, followed by oxygen tension and function. The latter two showed similar characteristics during stress, suggesting a greater role for oxygen tension in maintaining the electroretinogram as compared to blood flow. It is of interest that at an OPP of 0 mmHg there appears to be remnant responses. The following possibility may account for the preservation of ERG, LDF and pO2 at an OPP of 0 mmHg. The LDF receives contributions from both retinal and choroidal flows, thus when OPP = 0 mmHg the remnant flow may reflect choroidal flow. Zhi et al [56] have recently shown that retinal flow is more sensitive to IOP elevation than choroidal flow in rats. They also show that there is remnant choroidal flow at perfusion pressures very close to 0. As the photopic ERG b-wave reflects bipolar cell function and thus receives oxygen from both the inner and outer retinal blood supply, it is likely to be partially sustained by the choroid. Likewise, the preservation of oxygen may reflect choriocapillaris supply. An alternative explanation for the preservation of the ERG and pO_2_ is the introduction of exogenous metabolic substrates via air equilibrated Hanks balanced salt solution, which contains both oxygen and glucose. Exogenous metabolic substrate is highly likely to contribute to the preservations of the ERG at an ocular perfusion pressure of 0.

There were no differences in all three measures between 3- and 14-month-old rats during IOP elevation, suggesting that regulation of blood flow and oxygen in response to stress was unimpaired at 14 months of age. Despite these similarities age-related differences were observed during recovery.

During recovery from IOP elevation, ERG, LDF and vitreal pO_2_ show two distinct phases of recovery – a fast phase and a slower phase. This two-phased recovery characteristic was seen in all outcome measures, with the exception of LDF recovery in the young cohort, which showed a negligible second phase. The two phases are likely to reflect the close relationship between the delivery of metabolites such as glucose [57]–[60] and oxygen [28], [61]–[65] needed for the restoration of retinal function [66]–[70].

The finding that blood flow recovers faster than oxygen and function was not surprising, as the sudden release of the large pressure head resulting from acute IOP lowering (120 mmHg back to 10 mmHg) would be expected to produce rapid capillary filling [16]. The rapid recovery of blood flow has also been attributed to a build-up of vasodilatory mediators in the tissue during ischemia, causing vasodilation exceeding normal vessel tone upon IOP lowering [16], [30].

In this study, a lag was observed between the recovery of blood flow and oxygen tension (Figures 5A and B). Such a lag in the restoration of oxygen has been observed in cardiac tissue [71], skeletal muscle [72], the brain [73] and the eye [74]. One explanation for this observed lag is that rapid recovery of blood flow during the first minute results in a shortened mean capillary transit time (MCT). As the MCT of oxygen carrying erythrocytes [75]–[78] is approximately inversely related to oxygen extraction [79], [80], this environment is less conducive to oxygen exchange. As capillaries fill and MCT slows, oxygen transfer increases and thus vitreal oxygen tension gradients return to baseline (∼ 7 minutes, Figure 5B). We found that vitreal oxygen tension overshoots baseline during recovery, consistent with reports in cats [81], [82] and pigs [27]. The reason for this overshoot is not clear and requires further investigation.

Studies have shown close associations between oxygen and the ERG [28], [60], [64], [68], [83]–[86]. In this study, we show that whilst recovery of vitreal oxygen tension is faster than the ERG, the two outcome measures share similar phases (Figure 5B and C). We speculate that the lag between the ERG and oxygen tension might reflect a delay between the supply of oxygen and its utilisation. In addition to oxygen supply, glucose delivery is needed to provide substrate for the Kreb's cycle. Restoration of Kreb's cycle provides substrates needed for oxidative metabolism, glutamate production and neurotransmission, which ultimately restores the b-wave of the ERG. As the retina requires active glucose transporters to fuel the Kreb's cycle, there may be a lag before oxidative metabolism is restored, producing a relative surplus of free oxygen.

### Ageing effects on blood flow, oxygen and ERG recovery

The middle-aged group showed faster blood flow recovery in the first phase (T1_0.9_) and greater recovery amplitudes in the second phase when compared to the young group. This however did not translate to greater vitreal oxygen levels at the end of two hours when compared to the young (AE2), with both groups exhibiting a similar level of overshoot from baseline (19% to 34%). On the other hand, although ERG recovery was slightly faster during phase 1 in older eyes, at the end of 2 hours, older eyes showed less ERG recovery than did the younger group (86.9% versus 104.9%, respectively). Whether this translates to permanent functional loss needs clarification.

In middle aged animals, a second overshoot in blood flow was observed (Figure 5A). The reason for this is unclear. One idea is that older animals require high blood flow to deliver oxygen and to sustain retinal function, with greater reliance on constant delivery rather than high oxygen extraction. This was in contrast to the younger cohort which showed only one phase for blood flow recovery and negligible hyperperfusion, yet displayed an obvious hyper-oxygenation response over time. In addition to this, the older cohort showed slower recovery of oxygen tension relative to blood flow at the beginning, further implying poorer oxygen regulation. As argued above, this could be due to faster MCTs in older eyes, which may be detrimental to oxygen exchange. Another possibility is that oxygen consumption is greater in older eyes in order to support the ERG. This theory is supported by brain literature where Lu and colleagues showed that there is increased oxygen consumption with age per unit of brain volume [87]. They suggested that neuronal computational inefficiencies or leaky membrane ion channels result in the need for increased Na^+^K^+^-ATPase activity with age to maintain polarity. Increased oxygen consumption may also be caused by age-related mitochondrial dysfunction [3], [88], [89] that would lead to reduced ATP-production for a given oxygen load.

Perhaps the most interesting finding in this study was that despite normal oxygen tension recovery, the ERG in the older cohort did not recover fully after 2 hours when compared to their own baseline. In contrast, the younger cohort showed full recovery (Figure 5C). This finding is consistent with a previous study in ageing mice [3], [89] and lends further support to the idea that retinal neurons in ageing rats do not recover as well following stress [89], [90]. Whether retinal function in the middle-aged cohort has the capacity to fully recover to baseline with more time requires further investigation.

## Summary

Simultaneous real time measurement of relative ocular blood flow, oxygen tension and retinal function during IOP elevation supports the role of oxygen tension in the generation of retinal function, while autoregulation ensures a constant delivery of metabolic substrates required for normal functioning. Recovery from IOP stress uncovers heterogeneity in the temporal aspects of recovery. We show that blood flow recovers first, followed by oxygen tension within ten minutes. Retinal function recovered to only half of its baseline amplitude in the same time, showing a more prolonged recovery spanning the next hour. Interestingly all parameters reveal a two phased recovery. Middle-age rats show robust regulation of blood flow in both phases, effectively maintaining oxygen tension. Despite this, retinal function in the 14-month-old group did not recover fully. The nature and cause of this retinal functional deficit needs further investigation.

## Supporting Information

Figure S1 **Probe distance and backscatter.** The schematic in panel A shows the sensor when placed within the vitreous chamber. Panel B shows the increase in backscatter values (mean ± SEM, n = 4 adult Long-Evans rats) as the probe approaches the retina. The shaded grey box indicates the optimal backscatter range for LDF recording.
(TIF)

Figure S2 **Simultaneous tail cuff sphygmomanometry and femoral artery blood pressure measurement in rats.** Panel A shows mean arterial pressure (MAP), measured through a femoral artery cannula; plotted against systolic blood pressure (SBP) measured via tail cuff sphygmomanometry. Panel B shows femoral cannulation measured SBP plotted against tail cuff measured SBP. Readings were taken from 27 anaesthetised rats with various blood pressure profiles (hypotensive, N = 7, normotensive, N = 10, hypertensive, N = 10). Blood pressures were manipulated using intravenous infusion of sodium nitroprusside (50–200 µg/kg/min), saline control (normotensive group) and angiotensin-II (45–90 µg/kg/min) respectively. The data was fitted with a Deming regression (femoral MAP vs tail cuff SBP y = 0.91x+16.31, r2 = 0.62; femoral SBP vs tail cuff SBP y = 1.07x+5.29, r2 = 0.53). The analyses show good correlations between directly measured MAP/SBP and non-invasive SBP.
(TIF)

Figure S3 **Peripheral oxygen saturation in young Long Evans rats.** This figure shows the peripheral oxygen saturation (spO_2_) as measured in young Long Evans rats. Average starting spO_2_ (mean ± SD) was 93±4. SpO­_2_ remained constant over the three-hour period (one-way ANOVA, F = 0.55, P = 0.87). Oxygen saturation values were obtained using the Vetsens (Hornsby, NSW, Aust) P02 veterinary pulse oximeter.
(TIF)

Figure S4 **Backscatter in 3-month-old rats (N = 14) and 14-month-old (N = 16) measured using a combined LDF/PO2 probe.** Backscatter (error bars: SEM) was largely stable throughout the experiment as IOP was elevated in young (one-way ANOVA, F_19,218_ = 0.55, P = 0.94) and old (one-way ANOVA, F_19,252_ = 0.20, P>0.99) rats.
(TIF)
