# Supplementary figures and images for: The Effect of Ageing on Ocular Blood Flow, Oxygen Tension and Retinal Function during and after Intraocular Pressure Elevation
Source: PLoS One. 2014 May 27;9(5):e98393. doi: 10.1371/journal.pone.0098393 (PMC4035318; doi:10.1371/journal.pone.0098393)

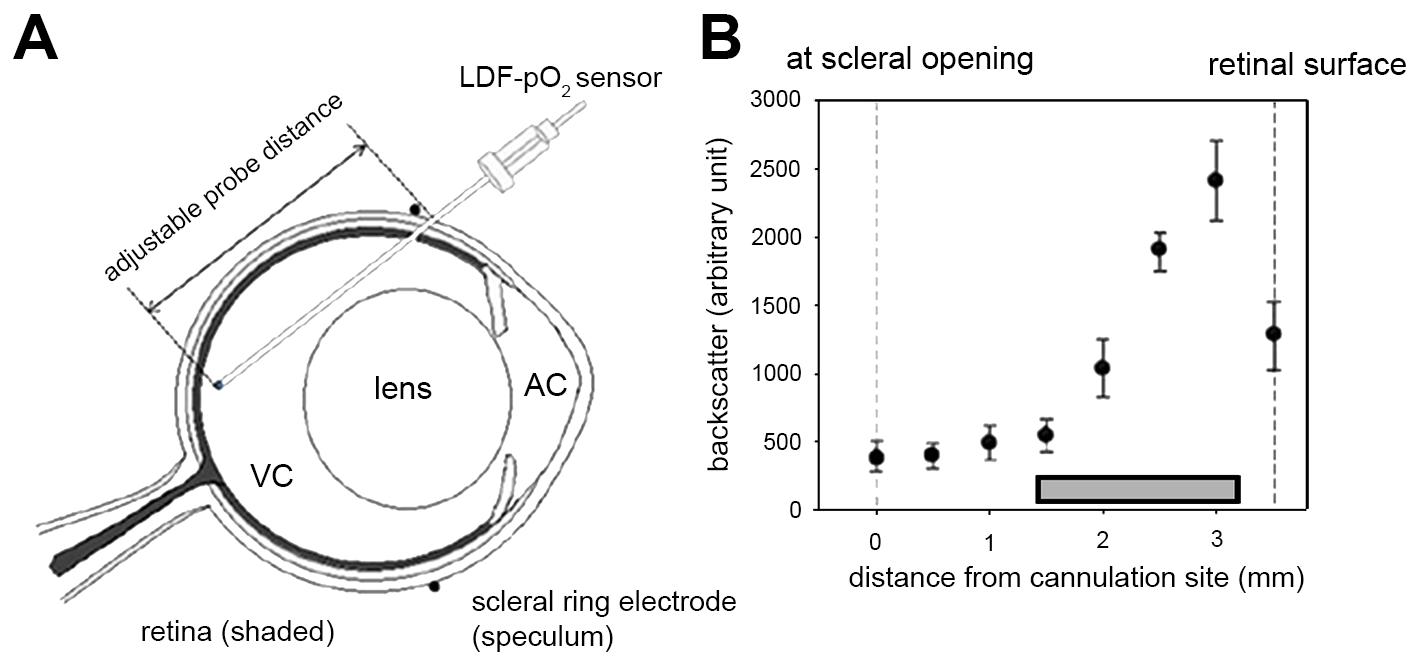

Supplement: Figure S1 — Probe distance and backscatter. The schematic in panel A shows the sensor when placed within the vitreous chamber. Panel B shows the increase in backscatter values (mean ± SEM, n = 4 adult Long-Evans rats) as the probe approaches the retina. The shaded grey box indicates the optimal backscatter range for LDF recording. (TIF) [file pone.0098393.s001.tif]

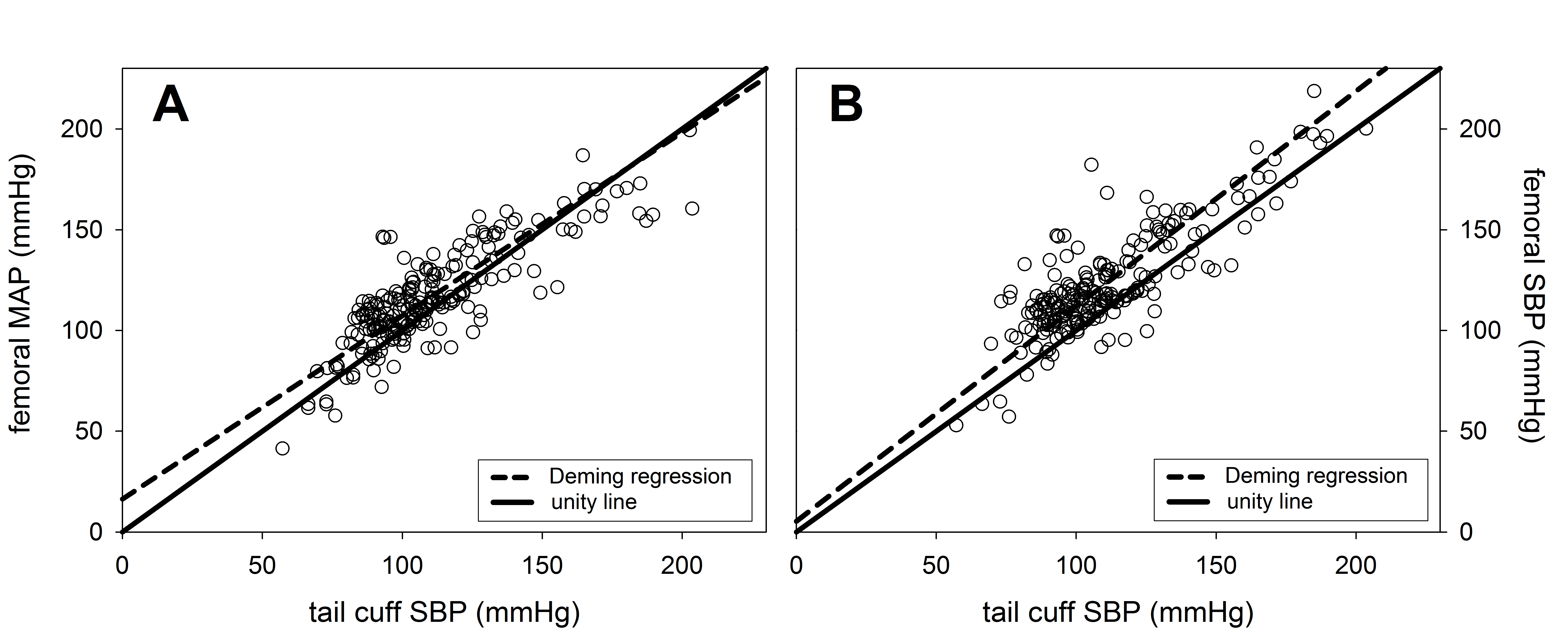

Supplement: Figure S2 — Simultaneous tail cuff sphygmomanometry and femoral artery blood pressure measurement in rats. Panel A shows mean arterial pressure (MAP), measured through a femoral artery cannula; plotted against systolic blood pressure (SBP) measured via tail cuff sphygmomanometry. Panel B shows femoral cannulation measured SBP plotted against tail cuff measured SBP. Readings were taken from 27 anaesthetised rats with various blood pressure profiles (hypotensive, N = 7, normotensive, N = 10, hypertensive, N = 10). Blood pressures were manipulated using intravenous infusion of sodium nitroprusside (50–200 µg/kg/min), saline control (normotensive group) and angiotensin-II (45–90 µg/kg/min) respectively. The data was fitted with a Deming regression (femoral MAP vs tail cuff SBP y = 0.91x+16.31, r2 = 0.62; femoral SBP vs tail cuff SBP y = 1.07x+5.29, r2 = 0.53). The analyses show good correlations between directly measured MAP/SBP and non-invasive SBP. (TIF) [file pone.0098393.s002.tif]

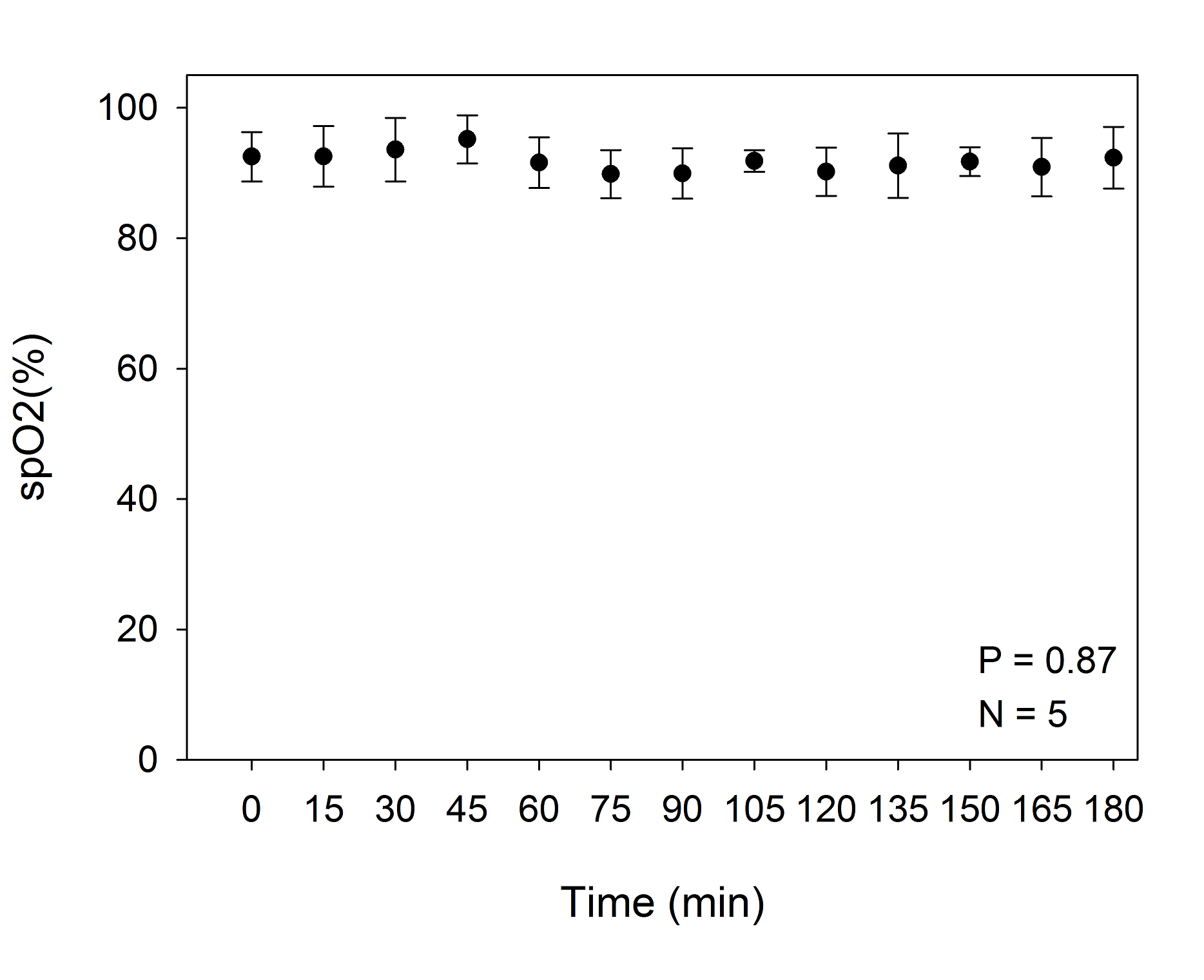

Supplement: Figure S3 — Peripheral oxygen saturation in young Long Evans rats. This figure shows the peripheral oxygen saturation (spO2) as measured in young Long Evans rats. Average starting spO2 (mean ± SD) was 93±4. SpO­2 remained constant over the three-hour period (one-way ANOVA, F = 0.55, P = 0.87). Oxygen saturation values were obtained using the Vetsens (Hornsby, NSW, Aust) P02 veterinary pulse oximeter. (TIF) [file pone.0098393.s003.tif]

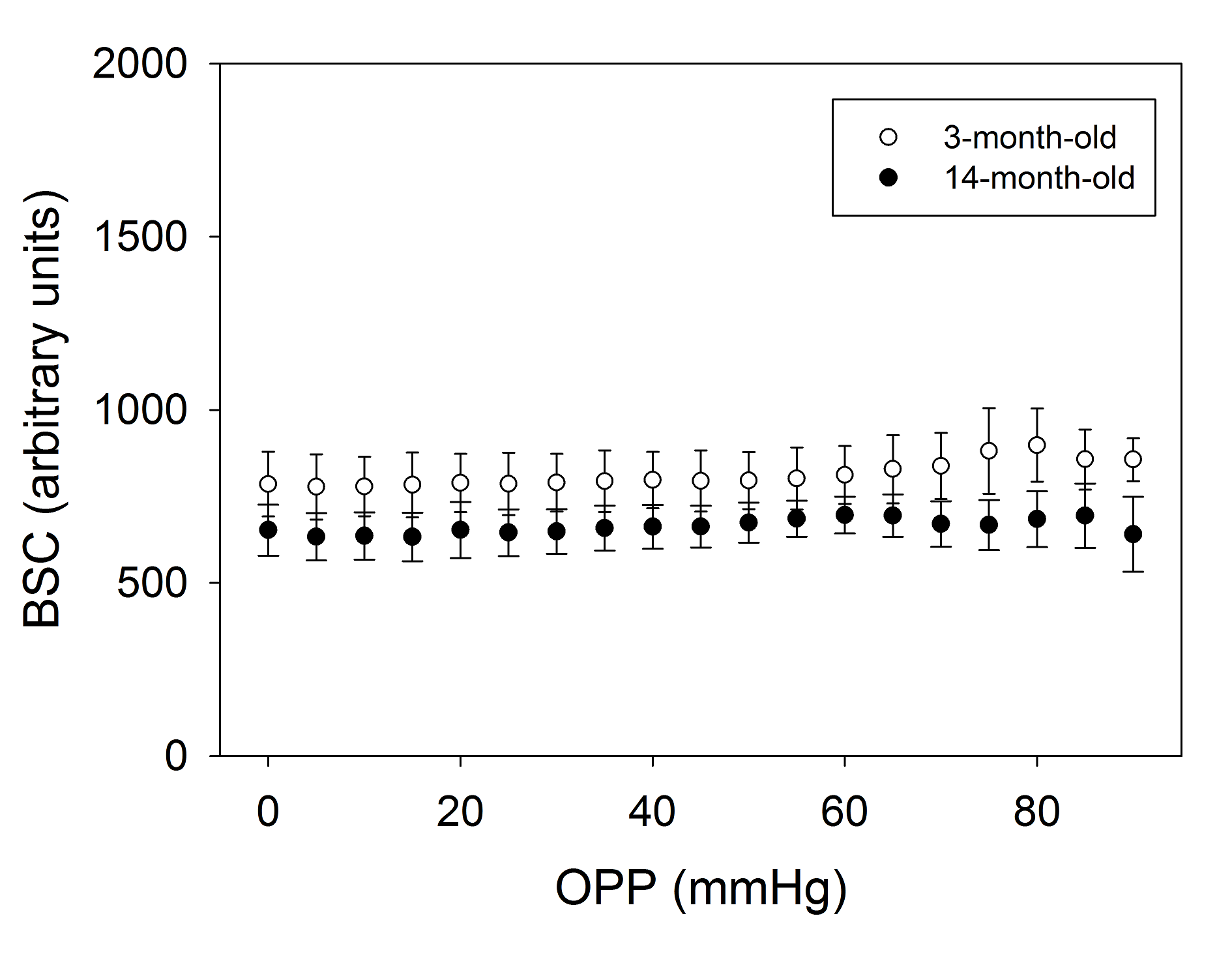

Supplement: Figure S4 — Backscatter in 3-month-old rats (N = 14) and 14-month-old (N = 16) measured using a combined LDF/PO2 probe. Backscatter (error bars: SEM) was largely stable throughout the experiment as IOP was elevated in young (one-way ANOVA, F19,218 = 0.55, P = 0.94) and old (one-way ANOVA, F19,252 = 0.20, P>0.99) rats. (TIF) [file pone.0098393.s004.tif]
